# Unraveling the significance of exosomal circRNAs in cancer therapeutic resistance

**DOI:** 10.3389/fphar.2023.1093175

**Published:** 2023-02-15

**Authors:** Fanhua Kang, Yuanliang Yan, Yuanhong Liu, Qiuju Liang, Zhijie Xu, Wei Zhu, Abhimanyu Thakur

**Affiliations:** ^1^ Department of Pathology, Xiangya Changde Hospital, Changde, Hunan, China; ^2^ Department of Pharmacy, Xiangya Hospital, Central South University, Changsha, Hunan, China; ^3^ National Clinical Research Center for Geriatric Disorders, Xiangya Hospital, Central South University, Changsha, Hunan, China; ^4^ Department of Pathology, Xiangya Hospital, Central South University, Changsha, Hunan, China; ^5^ Pritzker School of Molecular Engineering, Ben May Department for Cancer Research, University of Chicago, Chicago, IL, United States

**Keywords:** exosomes, extracellular vesicles, cancer, circular RNAs, drug resistance

## Abstract

Exosomes are nanoscale extracellular vesicles secreted by a variety of cells, affecting the physiological and pathological homeostasis. They carry various cargoes including proteins, lipids, DNA, and RNA and have emerged as critical mediators of intercellular communication. During cell–cell communication, they can internalize either by autologous or heterologous recipient cells, which activate different signaling pathways, facilitating malignant progression of cancer. Among different types of cargoes in exosomes, the endogenous non-coding RNAs, such as circular RNAs (or circRNAs), have gained tremendous attention for their high stability and concentration, playing promising functional roles in cancer chemotherapeutic response by regulating the targeted gene expression. In this review, we primarily described the emerging evidence demonstrating the important roles of circular RNAs derived from exosomes in the regulation of cancer-associated signaling pathways that were involved in cancer research and therapeutic interventions. Additionally, the relevant profiles of exosomal circRNAs and their biological implications have been discussed, which is under investigation for their potential effect on the control of cancer therapeutic resistance.

## 1 Introduction

Globally, cancers serve as one of the primary reasons for deaths, including in China (Bray et al., 2021), resulting in a serious economic and social burden (Stanway et al., 2021). Up to now, a variety of therapeutic strategies, such as chemotherapy (Okusaka and Furuse, 2020), immunotherapy (Sharma et al., 2017; Keenan and Tolaney, 2020), and targeted therapy (Yang et al., 2021a), have ameliorated the clinical treatment effects for cancer patients (Tsimberidou, 2015). However, due to their heterogeneity, cancer cells often exhibit primary or acquired treatment resistance, thereby resulting in treatment failure (Xu et al., 2020a; Liu et al., 2021a).

Exosomes are a subset of cell-derived nanovesicles with a diameter approximately ranging from 30 to 200 nm and participate in intracellular communication (Thakur et al., 2020a; Gaurav et al., 2021a; Thakur et al., 2021). They are released from almost all types of cells and remain more stable for a long time while participating in intracellular communication (Nie et al., 2021). During cell–cell communication, they can be internalized either by their own parent cells (referred as autologous uptake) or by different recipient cells (referred as heterologous uptake), which trigger different phenotypes in the recipient cells. Also, they can impersonate the constituents of their originating parent cells. This infers that the different cargoes from exosomes could be utilized as reliable prognostic and diagnostic biomarkers for multiple cancers (Thakur et al., 2017; Thakur et al., 2020b; Gaurav et al., 2021b). As one of the several different types of cargoes, circular RNAs (or circRNAs) have been found to interfere with the therapeutic response. A plethora of research studies have been conducted, which contributed toward the advancement of our knowledge about exosomal circRNAs (Hon et al., 2019; Wang et al., 2022a; Long et al., 2022).

Recently, circRNAs have emerged as promising regulatory factors for the malignant progression and treatment resistance (Liu et al., 2020; Yang et al., 2021b). In 1976, Sanger et al. used electron microscopy and preliminarily discovered the single-stranded closed-loop RNA molecules, circRNAs, in the virus (Sanger et al., 1976). Subsequent studies have pointed out that exosomes mainly comprise the following four categories, namely, circular intronic circRNAs, exon–intron circRNAs, exon circRNAs, and fusion circRNAs (Zhang et al., 2013; Guarnerio et al., 2016). Because of the deficiency of 5′ and 3′ terminals in structures, circRNAs are more stable than linear RNAs (Li et al., 2015). Studies have showed the important roles of aberrant circRNA-associated signaling in cancer pathogenesis and therapeutic response (Lai et al., 2021; Chen et al., 2022). Because of their unique closed-loop form and structural stability, circRNAs have been serving as novel prognostic and therapeutic targets for human cancers (Jiao et al., 2021; Lan et al., 2021).

Therapeutic resistance is one of the main challenges to cancer treatment (Wade and Kyprianou, 2018; Long et al., 2019). Because of the primary or acquired resistance to therapeutic agents, the overall therapeutic efficiency on cancer patients was limited, ultimately resulting in severe clinical and social problems. Meanwhile, the underlying molecular mechanisms for treatment resistance are varied and complex (Yan et al., 2019a; Yan et al., 2019b). Among these, the profiles of circRNAs have also been reported in exosomes, which can determine cancer heterogeneity and responses to therapeutic resistances. Apparently, in this review, we have discussed in detail about the different aspects of exosomal circRNAs in the context of drug resistance development, causing malignant progression of cancer.

## 2 Implication of exosomal *circRNAs* for cancer development and prognosis

### 2.1 Application of exosomal circRNAs as cancer biomarkers

**FIGURE 1:**
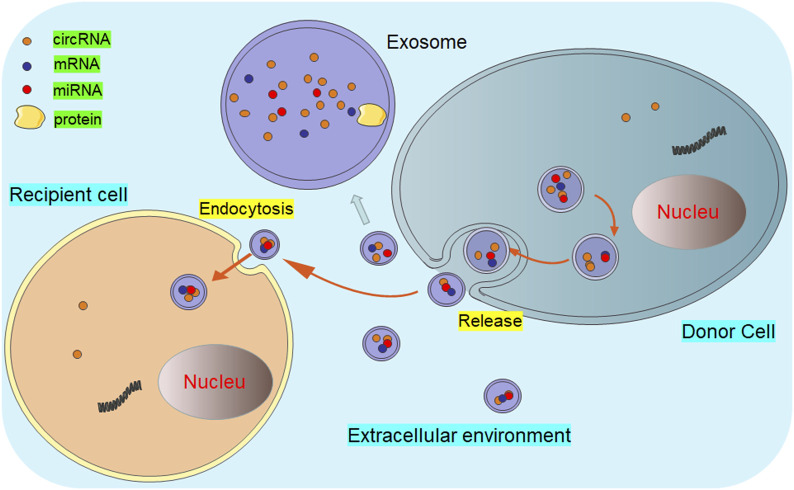
Diagram of exosomal circRNAs released from donor cells to recipient cells. The circRNA-containing exosomes are secreted into the extracellular environment by the donor cells. Thereafter, these exosomal circRNAs are transferred to the recipient cells through endocytosis or membrane fusion, executing multiple biological functions.

It is an important medical concern to explore the precise bio-targets for cancer treatment. Currently, some studies have showed the promising biological functions of non-coding RNAs in cancer pathogenesis and therapeutic response, such as microRNAs and long non-coding RNAs (Dumache, 2017; Zhao et al., 2018; Fadhil et al., 2020). As a new member of non-coding RNAs, circRNAs are released from almost all types of cells and could maintain the balance of body fluids (Figure 1). Customarily, exosomal circRNAs are balanced in the body fluids (Wang et al., 2019a; Verduci et al., 2021), participating in the pathogenesis of cancers (Zhang et al., 2018; Wu et al., 2022). Recently, Zhang et al. have evaluated the profiles of circRNAs in the colorectal cancer tissue. The results from this report demonstrated that the overexpression of circZNF609 could reduce cell proliferation and promote cell apoptosis by upregulating p53 expression (Zhang et al., 2019). Thus, exosomal circRNAs can be used as promising biomarkers for cancer development (Wang et al., 2020a; Wang et al., 2021a).

### 2.2 Early diagnosis of cancers *via* the detection of exosomal circRNAs

Due to the absence of obvious symptoms and lack of specific diagnosis markers at an early stage, cancer patients are commonly diagnosed with the advanced stage of the disease and miss the optimal therapeutic opportunities. Owing to several advantages, including their convenient availability in biofluids like blood, cerebrospinal fluid, urine, and saliva, and their ability to mimic the constituents of their parent cells, exosomes have been reported to be excellent nano-theranostic tools (Thakur et al., 2022a; Thakur et al., 2022b). A recent study by Wang et al. (2020b) has observed the significantly upregulated hsa_circRNA_002178 in plasma-derived exosomes from lung adenocarcinoma patients, and this serum exosomal circRNA could act as the potential biomarker for patients’ diagnoses. Tian et al. (2019) have confirmed that exosomal circRASSF2 can be effectively released into the serum from laryngeal squamous cell carcinoma patients. Exosomal circRASSF2 is significantly overexpressed in the serum and, hence, could be considered a novel diagnostic target for laryngeal squamous cell carcinoma patients. Furthermore, overexpression of exosomal hsa_circ_0004771 in the serum could effectively distinguish the cancer patients from the healthy populations, which indicated the possible diagnostic roles of serum exosomal hsa_circ_0004771 for the patients with colorectal cancer (Lasda and Parker, 2016; Pan et al., 2019).

### 2.3 Monitoring of cancer prognosis *via* the detection of exosomal circRNAs

Exosomes have been extensively studied as a potential tool for prognosis of different diseases, including cancer progression (Wang et al., 2019b; Zhou et al., 2021). This provides the ability to monitor the disease progression *via* a non-invasive technique. Among various cargoes, several circRNAs have also been detected in exosomes for disease prognosis purposes (Sun et al., 2021). The upregulated exosomal circPRMT5 in the urine and serum was found to be closely related to tumor progression of bladder urethral epithelial carcinoma patients. Also, exosomal circPRMT5 has been expected to be a non-invasive biomarker for the evaluation of prognostic values and therapeutic efficacy of patients with bladder urethral epithelial carcinoma (Chen et al., 2018). Moreover, survival analysis verified that pancreatic cancer patients with a high level of exo-circPDE8A displays unfavorable prognosis (Li et al., 2018). In addition, FLI1 exonic circRNAs, a newly identified oncogenic driver, could also serve as an unfavorable prognostic biomarker for small-cell lung cancer patients (Li et al., 2019). Taken together, these aforementioned studies suggested the functional roles of exosomal circRNAs as promising markers for cancer patients’ prognoses.

## 3 Exosomal circRNAs in cancer therapeutic resistance

### 3.1 Significance of exosomal circRNAs in chemotherapy resistance

**FIGURE 2:**
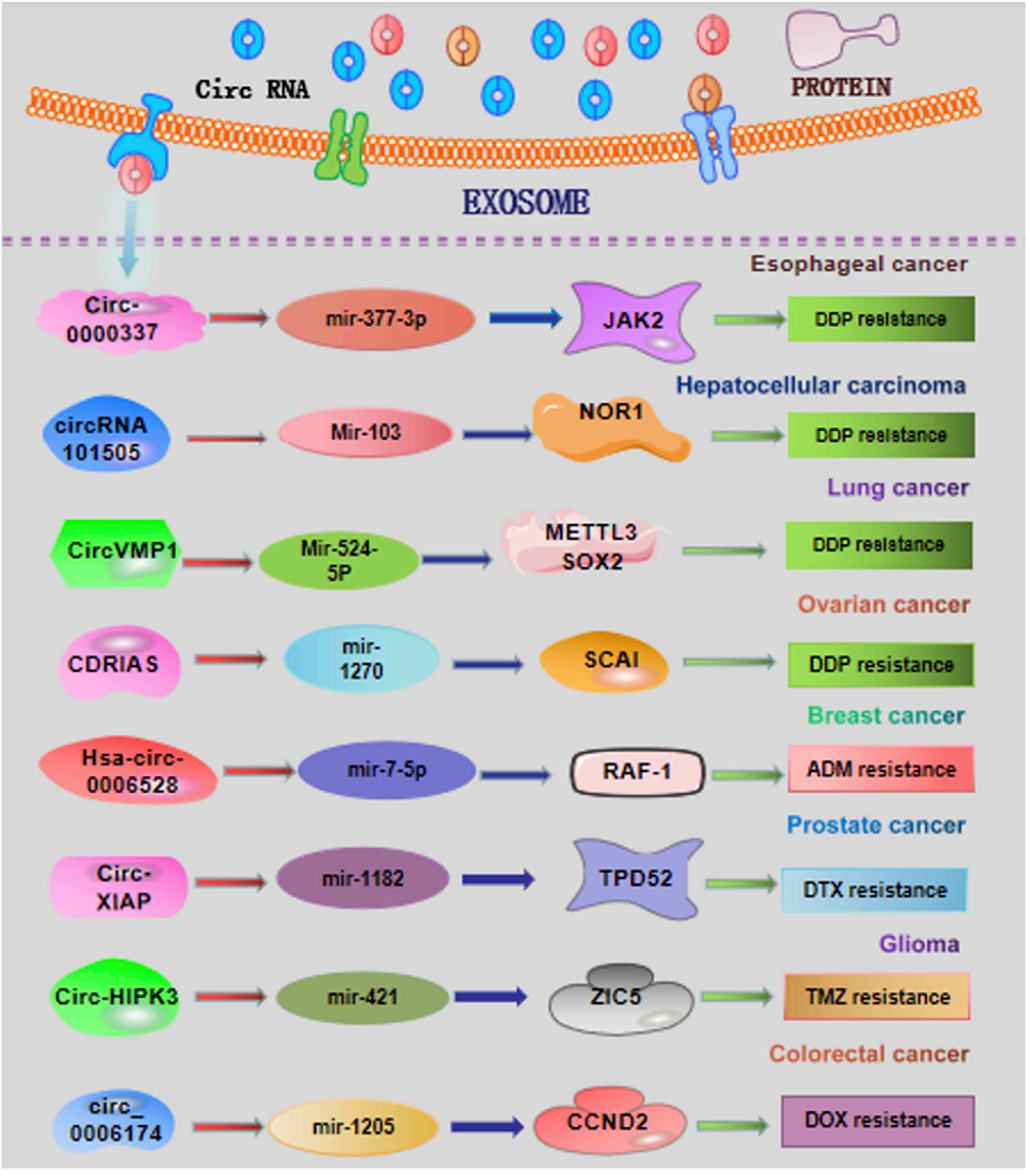
Overview of exosomal circRNAs in cancer chemotherapy resistance. DDP, cisplatin; ADM, Adriamycin; DTX, docetaxel; TMZ, temozolomide.

As one of the most effective strategies, chemotherapy is widely used for the clinical management of cancer patients (Carceles-Cordon et al., 2020). Recently, exosomes could transfer multiple circRNAs from donor cells to recipient cells, participating in the regulation of chemotherapeutic response (Figure 2). Emerging studies have implied that exosomal circRNAs are expected to become the bio-targets for the improvement of therapeutic efficacy.

#### 3.1.1 Esophageal cancer

Zang et al. (2021) have showed that circ-0000337 and JAK2 were highly expressed in cisplatin (DDP)-resistant esophageal cancer cells and tissues. The highly expressed circ-0000337 derived from exosomes obviously promotes cell proliferation and DDP resistance of esophageal cancer cells *in vitro* and *in vivo*. The exosomal circ-0000337 from DDP-resistant esophageal cancer cells could be transported into DDP-sensitive cells, consequentially resulting in therapeutic resistance. Mechanistically, serving as a sponge of miR-377-3p, exosomal circ-0000337 could promote the activation of JAK2 signaling, accelerating the DDP resistance (Zang et al., 2021).

#### 3.1.2 Hepatocellular carcinoma

CircRNA-101505 has recently been demonstrated to be downregulated in hepatocellular carcinoma (HCC) cells and tissues (Luo et al., 2019). Overexpression of circZFR displays the important inhibitory effects on cancer development and DDP chemotherapy. CircRNA_101505 could upregulate the expression of oxidized-nitro domain-containing protein 1 *via* sponging miR-103, facilitating the DDP sensitization of HCC patients (Luo et al., 2019). Zhou et al. found that cancer-associated fibroblasts (CAFs) could transfer circZFR into HCC cells, restraining the STAT3/NF-κB pathway and promoting HCC development and chemoresistance (Zhou et al., 2022).

#### 3.1.3 Lung cancer

Xie et al. have recently demonstrated the upregulated levels of exosomal circVMP1 in non-small cell lung cancer (NSCLC) cells. The findings in this study also revealed that circVMP1 expression was markedly elevated in DDP-resistant NSCLC cell lines A549/DDP and H1299/DDP (Xie et al., 2022). Acting as a ceRNA, circVMP1 could sponge microRNA-524-5p (miR-524-5p) to upregulate the expression of methyltransferase 3, the N6-adenosine-methyltransferase complex catalytic subunit (METTL3), and SOX2. CircVMP1 silencing significantly restrained the malignant behaviors and DDP resistance of A549/DDP and H1299/DDP cells.

#### 3.1.4 Ovarian cancer

Several studies have found the essential roles of exosomal circRNAs in influencing the drug sensitivity. The DDP sensitivity of ovarian cancer cells could be enhanced by upregulation of the exosomal circRNA Cdr1as (Sang et al., 2018; Zhao et al., 2019). The results from these studies reveal that exosomal circRNA Cdr1as could be transferred to the cancer cells and sponge miR-1270 to alleviate DDP resistance in ovarian cancer. Hence, it might be a hopeful strategy to sensitize the treatment response for cancer patients by regulating the release of exosomal circRNAs. Gao and Huang revealed that circ_0007841 induced cell apoptosis in DDP-resistant ovarian cancer cells by acting as a miR-532-5p sponge, consequently weakening the DDP resistance and malignant behaviors in DDP-resistant ovarian cancer cells (Gao and Huang, 2022).

#### 3.1.5 Breast cancer

Through exploring the profiles of circRNAs in Adriamycin (ADM)-resistant breast cancer cell MCF-7/ADM, Gao et al. identified the significantly upregulated hsa_circ_0006528 in MCF-7/ADM cells. Several algorithms, such as TargetScan and miRanda, indicated miR-7-5p as the potential target of hsa_circ_0006528. Thereafter, cellular function analysis displayed that hsa_circ_0006528 overexpression promoted the chemoresistance to ADM in breast cancer cells though sponging miR-7-5p (Gao et al., 2017). These results pointed out the important biological roles of circRNAs in chemo-resistance of breast cancer patients. Another recent study revealed that circRNA-CREIT was aberrantly downregulated in doxorubicin (DOX)-resistant triple-negative breast cancer (TNBC) cells and associated with a poor prognosis. Mechanistically, circRNA-CREIT can activate the RACK1/MTK1-mediated apoptosis signaling pathway. In addition, circRNA-CREIT could be packaged into exosomes and disseminate DOX sensitivity among TNBC cells (Wang et al., 2022b).

#### 3.1.6 Prostate cancer

A recent study has shown that knockdown of the exosomal circRNA X-linked inhibitor of apoptosis (circXIAP) could restrain cell migration and proliferation, and induce cell apoptosis, leading to docetaxel (DTX) sensitization in prostate cancer cells (Zhang et al., 2021). Downregulation of miR-1182 could effectively overturn the sensitization effects of circ-XIAP knockdown on DTX chemotherapy. Consequently, circ-XIAP derived from exosomes could enhance the therapeutic effects of DTX, offering predictive chemotherapy sensitizing targets for prostate cancer patients. Tan et al. revealed that circSFMBT2 was upregulated in DTX-resistant prostate cells and could increase the DTX resistance of prostate cells by regulating the miR-136-5p/TRIB1 axis (Tan et al., 2022).

#### 3.1.7 Glioma

Ding et al. observed that depletion of exosomal circNFIX increased temozolomide (TMZ) sensitization in glioma cells (Ding et al., 2020). This study proved that circNFIX transferred by exosomes could weaken the killing effect of TMZ on glioma cells by acting as a sponge of miR-132. Analogously, Han et al. demonstrated that aberrantly expressed exosomal circHIPK3 could accelerate the cancer progression and TMZ resistance by adjusting the miR-421/ZIC5 signaling axis (Han et al., 2021). Geng et al. demonstrated the exosome-mediated delivery of circWDR62 in TMZ-resistant glioma cells U251-R. High expression of exosomal circWDR62 can enhance TMZ resistance and malignant progression *via* regulating the miR-370-3p/MGMT axis (Geng et al., 2022).

#### 3.1.8 Colorectal cancer

A recent study demonstrated that the circ_0006174 derived from exosomes was significantly upregulated in DOX-resistant colorectal cancer tissues and cells (Zhang et al., 2022). This research found that the effect of exosomal circ_0006174 on DOX resistance was dependent on the miR-1205-mediated CCND2 upregulation. Li et al. found that exosome-derived circ_0094343 was remarkably downregulated in chemotherapy-resistant colorectal cancer tissues and metastatic colorectal cancer tissues (Li and Li, 2022). Mechanistic validation demonstrated that circ_0094343 could inhibit HCT116 cell proliferation and improve the therapeutic resistance to 5-fluorouracil (5-FU), oxaliplatin, and DOX *via* the miR-766-5p/TRIM67 axis.

### 3.2 Implication of exosomal circRNAs in immunotherapeutic resistance

**FIGURE 3:**
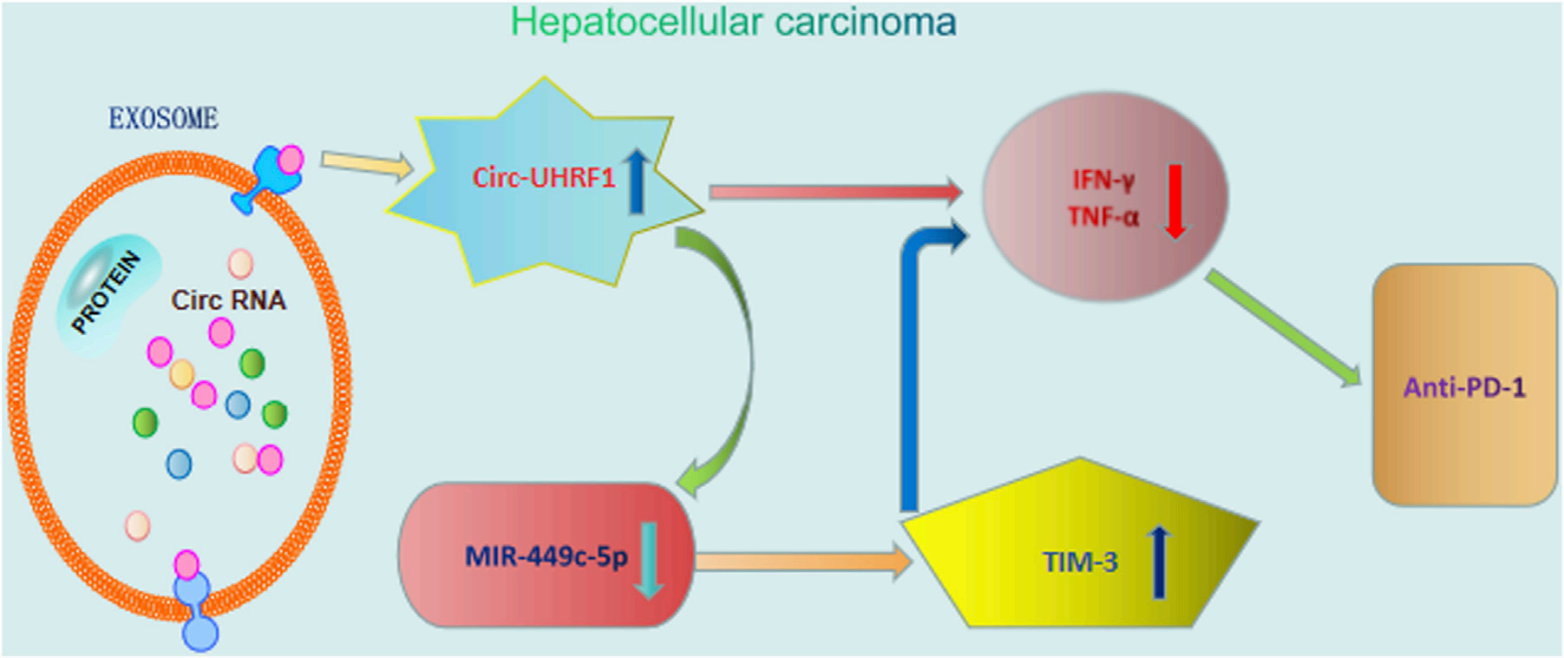
Roles of exosomal circRNAs in immunotherapy of hepatocellular carcinoma. Exosomal circ-UHRF1 released from HCC cells effectively inhibits the function of natural killer cells by degrading miR-449c-5p and inducing TIM-3 expression, finally reinforcing anti-PD1 immunotherapy.

Nowadays, immunotherapy is becoming an attractive therapeutic method that helps in effectively resisting the cancer cells by ameliorating the host immune response. Targeting the host immune system could initiate a systemically permanent anti-tumor immune response. Despite the unprecedented tumor regressions and long-term survival benefits observed with anti-programmed death 1 (anti-PD1) therapy in patients with advanced cancers, there is still a subset of patients who do not obtain a curative benefit from immunotherapy (Topalian et al., 2020). Recently, exosomal circRNAs have been proved to affect the therapeutic response to anti-PD1 and provided hopeful biomarkers for improving immunotherapy. Accordingly, circ-UHRF1 knockdown in HCC cells could sensitize the anti-PD1 treatment, consequently ameliorating the patients’ survival rate (Zhang et al., 2020). In addition, exosomal circ-UHRF1 released from HCC cells effectively inhibits the function of natural killer cells by degrading miR-449c-5p and inducing TIM-3 expression, finally reinforcing the immunosuppressive tumor microenvironment (Huang et al., 2020) (Figure 3).

### 3.3 Involvement of exosomal circRNAs in targeted therapy resistance

It has been suggested that cancer cells might become resistant to targeted therapy-mediated cellular cytotoxicity, interfering with the clinical therapeutic effects (Table 1). For example, Yang et al. found the upregulated circFN1 in both sorafenib-resistant HCC cells and tissues (Yang et al., 2020). Overexpression of circFN1 may function as a sponge for miR-1205 to facilitate sorafenib resistance and tumorigenesis of HCC cells.

**TABLE 1:** Various functions of circRNAs in cancer drug resistance.

CircRNA	Expression	Drug response	Cancer	Reference
circ_0000337	Upregulation	Promoting cisplatin resistance	Esophageal cancer	Zang et al. (2021)
circRNA_101505	Downregulation	Promoting cisplatin resistance	Hepatocellular carcinoma	Luo et al. (2019)
circZFR	Upregulation	Promoting cisplatin resistance	Hepatocellular carcinoma	Zhou et al. (2022)
circVMP1	Upregulation	Promoting cisplatin resistance	Lung cancer	Xie et al. (2022)
circRNA Cdr1as	Upregulation	Inhibiting cisplatin resistance	Ovarian cancer	Sang et al. (2018), Zhao et al. (2019)
circ_0007841	Upregulation	Promoting cisplatin resistance	Ovarian cancer	Gao and Huang (2022)
hsa_circ_0006528	Upregulation	Promoting Adriamycin resistance	Breast cancer	Gao et al. (2017)
circRNA-CREIT	Downregulation	Promoting doxorubicin resistance	Breast cancer	Wang et al. (2022b)
circXIAP	Downregulation	Inhibiting docetaxel resistance	Prostate cancer	Zhang et al. (2021)
circSFMBT2	Upregulation	Promoting docetaxel resistance	Prostate cancer	Tan et al. (2022)
circNFIX	Downregulation	Inhibiting temozolomide resistance	Glioma	Ding et al. (2020)
circHIPK3	Upregulation	Promoting temozolomide resistance	Glioma	Han et al. (2021)
circWDR62	Upregulation	Promoting temozolomide resistance	Glioma	Geng et al. (2022)
circ_0006174	Upregulation	Promoting docetaxel resistance	Colorectal cancer	Zhang et al. (2022)
circ_0094343	Downregulation	Promoting 5-fluorouracil oxaliplatin and docetaxel resistance	Colorectal cancer	Li and Li (2022)
circUHRF1	Downregulation	Inhibiting anti-PD1	Hepatocellular carcinoma	Zhang et al. (2020)
circFN1	Upregulation	Promoting sorafenib resistance	Hepatocellular carcinoma	Yang et al. (2020)
circRNA-SORE	Downregulation	Inhibiting sorafenib resistance	Hepatocellular carcinoma	Xu et al. (2020b)

Similarly, circRNA-SORE transported by exosomes plays a promoting role in sorafenib resistance in HCC (Xu et al., 2020b). Depletion of circRNA-SORE could enhance the cell-killing ability of sorafenib through improving the stability of the oncogenic protein YBX1.

### 3.4 Importance of exosomal circRNAs in radiotherapy resistance

He et al. (2022) demonstrated that exosomal circPRRX1 affected cell proliferation, motility, invasion, and radiation sensitivity *in vitro* and *in vivo*. Mechanistically, exosomal circPRRX1 affected the cancer-associated biological functions through regulating miR-596 and its downstream target NKAP (He et al., 2022). This study demonstrated the important roles of the exosomal circRNA–miRNA ceRNA crosstalk in adjusting tumorigenesis and radiation sensitivity. Another group’s study showed that low-dose radiation-induced exosomal circ-METRN played an oncogenic role in glioblastoma progression and radioresistance through the miR-4709-3p/GRB14/PDGFRα pathway (Wang et al., 2021b), providing novel mechanistic insights into the potential roles of exosomal circRNAs as therapeutic targets in glioblastoma.

## 4 Discussion and perspective

Because of the stable structures and attractive biological functions (Huang et al., 2021; Papaspyropoulos et al., 2021), circRNAs are intrinsically resistant to degradation mediated by RNA exonuclease or RNase R and easily transferred into the body fluids by exosomes (Burd et al., 2010). More important, exosome-carried circRNAs have been proved to be the candidate non-invasive biomarkers for the diagnosis and prognosis of cancer patients (Wang et al., 2021c; Li et al., 2021). Meanwhile, exosomal circRNAs, as available therapeutic targets, have also been obtained for clinical practice (Harper et al., 2021).

The preexisting drug-resistant cells and the continuous self-differentiation of cancer cells lead to the accumulated therapy-resistant characteristics of cancer patients (Haider et al., 2020). Cancer-related exosomes, released by cancer cells and other cells in the tumor micro-environment, play regulatory roles in the progression, migration, and deterioration of multiple cancers (Sandua et al., 2021). CircRNAs can be a novel type of therapeutic targets, by functioning as tumor suppressors or oncogenes (He et al., 2021; Pan et al., 2023). Accumulated studies have revealed that cancer cells could release circRNAs to the surrounding recipient cells *via* exosome-mediated transport strategies, thus affecting the carcinogenesis and therapeutic response (Liu et al., 2021b; Fontemaggi et al., 2021; Zhang et al., 2023). For example, the synthetic tumor-suppressing circRNAs can be packaged into exosomes and transported into certain target cells to suppress cancer metastasis and invasion (Dai et al., 2020). The circRNA-based research field has identified the vital functional roles of exosomal circRNAs in cancer biology (Harper et al., 2021). They could act as the pivotal biomarkers for early cancer detection, diagnosis, prognosis, and evaluation of therapeutic efficacy.

## 5 Conclusion

Cancers are the highest malignant diseases, resulting in the leading cause of death worldwide. Therapeutic resistance limits the effectiveness of therapies, presenting a major challenge for the clinical treatment of cancer patients. Exosomal circRNAs are recently demonstrated to regulate the response to therapeutic strategies. As biological delivery carriers, exosomes are of prodigious interest in cancer research and treatment. Nevertheless, the detailed mechanisms underlying the regulation and biological functions of exosomal circRNAs remain to be further explained. Therefore, clarifying the underlying molecular mechanisms regulated by the exosome-containing circRNAs may help strengthen the therapeutic sensitivity by modulating the tumor microenvironment.
